# Paediatric imaging radiation dose awareness and use of referral guidelines amongst radiology practitioners and radiographers

**DOI:** 10.1007/s13244-015-0449-2

**Published:** 2015-11-12

**Authors:** Jonathan L. Portelli, Jonathan P. McNulty, Paul Bezzina, Louise Rainford

**Affiliations:** Department of Radiography, Faculty of Health Sciences, University of Malta, Msida, Malta; Diagnostic Imaging, School of Medicine and Medical Science, University College Dublin, Dublin, Ireland

**Keywords:** Radiation dose awareness, Paediatrics, Diagnostic Imaging, Radiology, Radiography

## Abstract

**Objectives:**

The objectives are to investigate radiology practitioners’ and radiographers’ radiation dose awareness and use of referral guidelines for paediatric imaging examinations.

**Methods:**

A prospective cross-sectional survey was conducted amongst radiology practitioners and radiographers working at a primary paediatric referral centre in Malta. Part of the survey asked participants to indicate the typical effective dose (ED) for several commonly performed paediatric imaging examinations, answer five true-false statements about radiation protection principles, and specify their use of referral guidelines for paediatric imaging.

**Results:**

The return of 112 questionnaires provided a response rate of 66.7 %. Overall, imaging practitioners demonstrated poor awareness of radiation doses associated with several paediatric imaging examinations, with only 20 % providing the correct ED estimate for radiation-based examinations. Nearly all participants had undertaken radiation protection training, but the type and duration of training undertaken varied. When asked about the use of referral guidelines for paediatric imaging, 77.3 % claimed that they ‘did not’ or ‘were not sure’ if they made use of them.

**Conclusions:**

Poor awareness of radiation doses associated with paediatric imaging examinations and the non-use of referral guidelines may impede imaging practitioners’ role in the justification and optimisation of paediatric imaging examinations. Education and training activities to address such shortcomings are recommended.

**Key Points:**

• *Imaging practitioners demonstrated poor radiation dose awareness for 5 paediatric imaging examinations*.

• *Most radiology practitioners and radiographers were ‘not sure’ or ‘did not’ use referral guidelines*.

• *Imaging practitioners generally considered previously undertaken paediatric imaging examinations*.

• *Some imaging practitioners had not undertaken training in radiation protection for 10 years*.

• *Training activities to address imaging practitioners’ poor radiation dose awareness are encouraged*.

## Introduction

Medical imaging (MI) is an essential tool in clinical medicine that provides great benefits and may even be lifesaving [1]. However, since most MI examinations involve an exposure to ionising radiation, there are growing concerns that the increased demand and use of MI may cause late adverse effects, particularly when performed in young patients. Indeed, findings of two large long-term follow-up studies have indicated a small increased risk for the incidence of brain cancer and leukaemia in patients who underwent a CT when they were very young [2, 3]. Consequently, while there is considerable uncertainty and debate about the actual lifetime cancer risks associated with low dose radiation exposure (<100 mSv), such risks are still estimated to be between 2 and 5 times higher for paediatric patients when compared to adults [4–6].

It is important that imaging practitioners, particularly radiologists and radiographers, fulfil their responsibilities in maximising the benefit-to-risk ratio for each medical radiation exposure, as stipulated by national and international regulations [7, 8]. Therefore, apart from confirming that each MI examination requested is the most responsive and appropriate examination for the child at that point in time (justification), it is equally important that imaging practitioners ‘child-size’ (optimise) each MI examination according to the child’s physical characteristics and/or underlying clinical indications [9–12].

Radiologists and radiographers, therefore, need to be fully knowledgeable about the MI procedures they perform in order to be able to truly fulfil these important roles as effectively as possible. However, despite the numerous awareness campaigns and resources available, research evidence continues to suggest that health professionals, including radiologists and radiographers, generally have a poor level of awareness concerning radiation doses and risks of commonly performed MI examinations [13–24]. Furthermore, while this lack of knowledge may possibly be compensated by the use of referral guidelines, research also suggests that these are not widely used [25, 26]. While such findings do not necessarily mean that health professionals have inadequate understanding or competence in their work practices, questions arise as to what effect this gap in knowledge can have in the justification and optimisation of MI examinations.

Given the lack of research concerning radiation dose awareness amongst radiology practitioners and radiographers within Europe, and that the majority of previous studies had mainly focused on adult MI examinations, the authors sought and obtained ethical approval from the governing institution to conduct this prospective cross-sectional survey at a large general hospital in Malta, which also serves as a primary referral centre for paediatric patients [27]. In view of the limited literature available, this study was performed to yield an important insight into the level of awareness and practice aspects amongst local radiology practitioners and radiographers with respect to MI examinations performed on paediatric patients. Consequently, the aims of the study being considered in this article were to investigate radiology practitioners’ and radiographers’ (i) level of awareness of radiation doses associated with some paediatric MI examinations; and (ii) their use of referral guidelines for paediatric MI examinations.

## Materials and methods

Following an extensive literature review, it was evident that no standardised tool existed to assess radiation dose awareness amongst health professionals. Nonetheless, while most research studies made use of a written questionnaire designed by its authors, the type of questions asked were generally similar in nature. Indeed, in several studies of radiation dose awareness were assessed by a specific section, which asked respondents to indicate the estimated effective dose (ED) range or equivalent number of chest radiographs typically associated with several commonly performed MI examinations [14–23].

The questionnaire in this study contained questions similar to those in past publications, but also incorporated further sections that addressed the aims being considered in this article, as well as other aims that will be discussed in subsequent publications. Most of the questions were closed ended and offered participants a list of possible pre-defined answers, although it was also possible for participants to elaborate on those provided or add their own response. In actual fact, two versions of this questionnaire were designed so that they could be respectively addressed to radiology practitioners and radiographers. The sole difference was that the questionnaire addressed to radiology practitioners asked about *‘performed and/or reported’* paediatric MI examinations while that addressed to radiographers asked about *‘performed’* MI examinations, since the latter do not report paediatric MI examinations at the hospital studied.

One section of the questionnaire required participants to indicate the typical estimated ED range generally associated with a paediatric CT of the head (5 year old), thorax (5 year old), and abdomen (1 year and 5 year old), together with the ED associated with a fluoroscopically-guided coronary angiography intervention performed on a paediatric patient. An MRI and an ultrasound scan were also included in this list to assess whether participants were aware that these examinations are non-ionising and, therefore, do not involve a radiation dose. Participants were expected to select the most appropriate ED in millisieverts (mSv) for the given examination, which included: 0, 0 to <0.03, 0.03 to <3, 3 to <6, 6 to <10, 10 to <30, and more than 30. A ‘Don’t know’ option was also included so as to enhance the validity and truthfulness of the answers obtained, as this option did not force participants to respond to a question they truly did not know [28]. Furthermore, an example clearly indicating that an adult postero-anterior (PA) chest radiograph was generally associated with an ED ranging from >0 to 0.03 mSv was provided. The expected answers to these examinations were based on information provided by the relevant literature [29–33]. Five true-false statements relating to basic radiation protection principles, medical exposure regulations, and radiation risks for paediatric patients were also included so as to allow for the evaluation of participants’ knowledge of such concepts. A further section included several questions relating to the use of referral guidelines and whether participants would generally consider previous MI examinations undertaken by the child, prior to pursuing with the requested MI examination.

The questionnaire’s reliability was measured through the test re-test method, whereby six participants form the target population that completed the questionnaire on two occasions, two weeks apart. The resultant mean reliability intraclass correlation co-efficient (ICC) obtained was that of 0.948, with the 95 % confidence interval ranging from 0.871 to 0.999. The content validity of the questionnaire was assessed by three experts (an experienced academic, a radiologist and a radiographer), who independently rated the relevance of each item against the study aims, resulting in a mean content validity index of 0.99. In its final form, the questionnaire consisted of a total of 20 questions and was estimated to take about 10 minutes to complete.

In order to facilitate the distribution of questionnaires to all imaging professionals, a list of radiology practitioners and radiographers providing MI services at the hospital being studied was sought and obtained by the authors. ‘Radiology practitioners’ in this study applied to radiologists, nuclear medicine physicians, and trainee radiology residents. From the list of imaging professionals provided, sixteen were excluded since they were either away on long leave or else they solely worked in mammography and, therefore, did not perform MI examinations on paediatric patients. The primary author personally met with each radiology practitioner and radiographer, invited them to participate in the study, and provided them with the questionnaire and an information letter. This information letter briefly explained the purpose of the study, emphasised the importance of a truthful response, and that participation was voluntary. It also assured anonymity of responses obtained and instructed willing participants to submit their completed questionnaires in one of the collection boxes provided within the MI department. In total, 168 questionnaires were collectively distributed to radiology practitioners (22) and radiographers (146) during the first week of July 2014. Another five radiology practitioners were not willing or were unable to participate in the study, citing that they did not have the time or else that they were not involved in any aspect related to paediatric imaging. All collection boxes were collected on the 1st of August 2014, allowing for a data collection period of approximately four weeks. Data was inputted into IBM SPSS version 20 (IBM Corporation, New York, USA) and statistical guidance from an experienced statistician was sought for its analysis. The Chi squared (*χ*^2^) test was used to explore possible associations between the responses provided by the two professions, while the Mann Whitney test was used to compare the mean ‘correct response score’ of both independent groups (since Kolmogorov-Smirnov *p* < 0.000; Shapiro-Wilk *p* = 0.002 determined that the score distribution was not normal). For all tests, the overall value for statistical significance was *P* < 0.05.

## Results

A total of 112 questionnaires were returned from the 168 distributed, resulting in an overall response rate of 66.7 %, consisting of 12 radiology practitioners (consultant/specialist radiologists/nuclear medicine physicians {*n* = 7}, trainee radiology residents {*n* = 5}) and 100 radiographers. The participant demographics are summarised in Table 1, with the majority of participants being female (58.0 %) and aged 35 years or younger (77.7 %). While 13.0 % had more than 21 years of clinical experience, the majority of participants (69.6 %) indicated that they had worked for 10 years or less.

**Table 1:** Summary demographics of study participants (% values in parenthesis)

Characteristics	Radiology practitioners (*n* = 12)	Radiographers (*n* = 100)
Female gender, *n* (%)	5 (41.7)	60 (60.0)
Age, *n* (%)		
<25 years	0	23 (23.0)
26–35 years	7 (58.3)	57 (57.0)
36–45 years	0	9 (9.0)
46–55 years	2 (16.7)	9 (9.0)
>56 years	3 (25.0)	1 (1.0)
Clinical experience, *n* (%)		
<2 years	2 (16.7)	17 (17.0)
3–10 years	5 (41.7)	54 (54.0)
11–20 years	0	20 (20.0)
>21 years	5 (41.7)	9 (9.0)
Education/training received in radiation protection	12 (100.0)	98 (98.0)
Lectures as part of undergraduate studies	2 (16.7)	84 (84.0)
Lectures as part of postgraduate studies	8 (66.7)	31 (31.0)
Attendance to conference/seminar/workshop	5 (41.7)	46 (46.0)
Attendance to radiation safety course	4 (33.3)	10 (10.0)
Induction training abroad	1 (8.3)	0
Own research	1 (8.3)	0
Hours of radiation protection education/training		
<1 h	0	0
2–10 h	6 (54.5)	29 (31.2)
11–20 h	0	21 (22.6)
21–30 h	3 (27.2)	13 (14.0)
31–50 h	2 (18.2)	17 (18.2)
>51 h	0	13 (14.0)
Time since last education/training		
<5 years	8 (66.7)	61 (62.3)
6–10 years	1 (8.3)	25 (25.5)
>10 years	3 (25.0)	12 (12.2)

Percentage values are based on number of responses obtained for each particular question

With the exception of two radiographers, all participants indicated that they had received education and/or training in radiation safety, mostly during their undergraduate and/or postgraduate training. Nearly half the radiology practitioners and radiographers also attended a conference, seminar, or workshop about radiation protection post qualification. While the perceived amount of training hours varied across the study sample, the majority did report that they had last received such education/training in the past 5 years.

### Awareness of radiation doses of paediatric imaging examinations

**Fig. 1:**
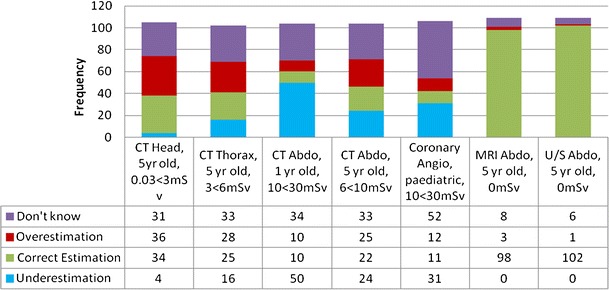
Summary of radiology practitioners’ and radiographers’ responses indicating the effective dose (ED) associated with seven paediatric MI examinations. The ED estimate considered as the correct response is also provided

**Fig. 2:**
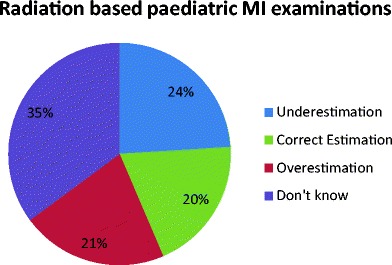
Overview of radiology practitioners’ and radiographers’ overall level of awareness concerning the ED associated with the five radiation based MI examinations

Consistent with findings of previous research, the overall findings of our study suggest that the majority of local radiology practitioners and radiographers were not aware of the typical ED range associated with common paediatric MI examinations (Fig. 1). Furthermore, if one had to solely consider the five radiation-based paediatric MI examinations; only 20 % of respondents indicated the correct ED range for such examinations, while 21 and 24 %, respectively, underestimated or overestimated the ED for these examinations (Fig. 2). Consequently, more than a third of participants (35.1 %) actually indicated that they ‘did not know’ the answer. Furthermore, while it was expected that the majority would know that MRI and ultrasound are not associated with a radiation exposure, it was surprising to note that four radiographers actually attributed an ED to these examinations (1.8 %, 4/218), with two radiology trainees and 12 radiographers (6.4 %, 14/218) even indicating that they ‘did not know’ that the ED for such examinations was 0 mSv.

### Awareness of radiation protection principles and increased risk for paediatric patients

The majority of radiology practitioners and radiographers (57.1 %, 64/112) correctly answered all five true-false statements concerning basic radiation protection principles, with another 31.3 % (35/112) only getting one response incorrect (Table 2). Nonetheless, there were still a number of participants whose responses indicated that they were not aware of the concepts of radiation safety principles such as justification and optimisation, as well as the increased lifetime cancer risk per unit dose of radiation for paediatric patients. In addition, it was evident that some respondents were unaware that each radiation exposure is believed to be cumulative and, therefore increases, a patients’ lifetime cancer risk.

**Table 2:** Summary of correct responses obtained for the five true-false statements relating to radiation protection principles, regulations, and potential for increased radiation risk for paediatric patients

True–False statements	Answer	Correct responses, *n* (%)	
		Radiology practitioners (*n* = 12)^a^	Radiographers (*n* = 100)^a^
The benefit of performing a medical imaging examination should be similar to the associated risks involved	False	11 (91.7)	84 (85.7)
It is estimated that paediatric patients have a 2 to 5 times higher lifetime cancer risk per unit dose of radiation when compared to adults	True	10 (90.9)	81 (83.5)
Maltese and European legislation stipulate that each medical exposure to ionising radiation must be justified	True	12 (100.0)	99 (99.0)
Optimisation refers to the principle by which each medical radiation exposure must provide the best image quality for diagnosis, irrespective of the radiation dose involved	False	9 (75.0)	95 (95.0)
Every single exposure to ionising radiation is cumulative and therefore increases an individual’s lifetime cancer risk	True	10 (83.3)	84 (84.0)

^a^Since some respondents chose not to answer particular questions, percentages are based on total number of responses obtained

### Correct responses score

**Fig. 3:**
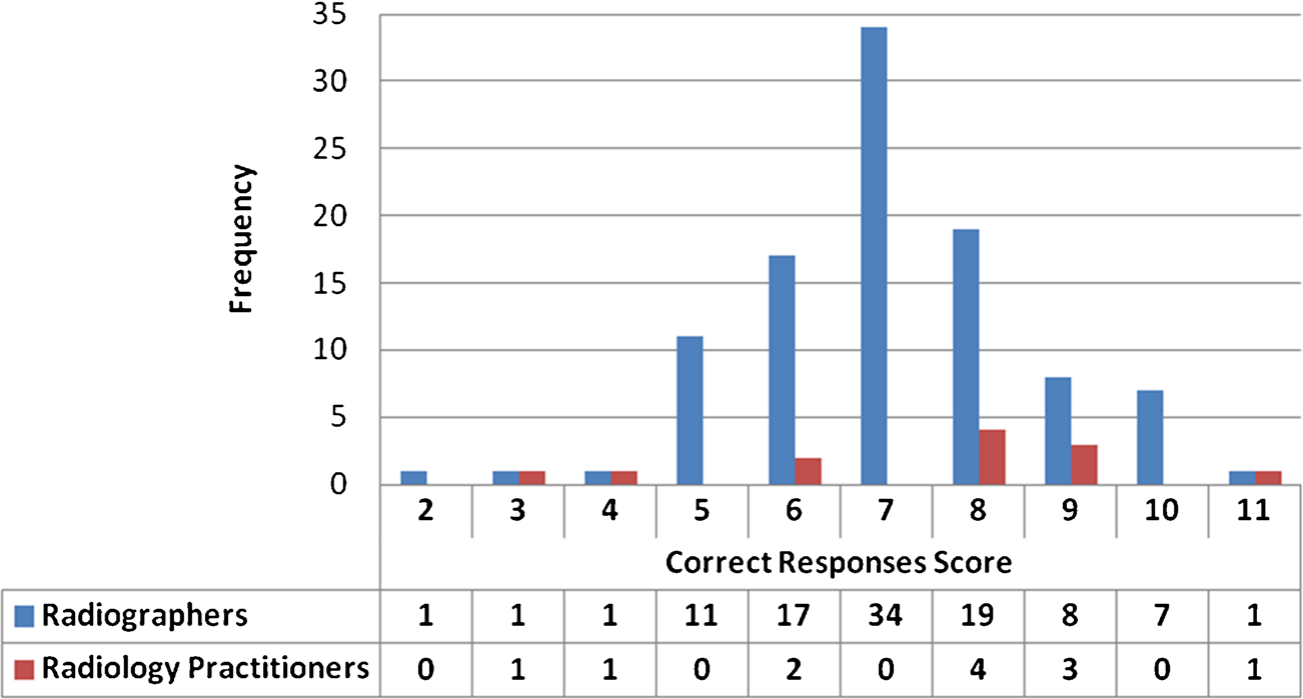
Correct responses score obtained by radiology practitioners and radiographers, with a mark of 12 being the maximum that could be achieved

While we acknowledge that the small number of radiology practitioners may have limited our analysis, only one statistically significant difference was found in the responses provided by the two different professions for the seven ED estimates or the five true-false statements. This related to the true-false statement concerning optimisation (*χ*^2^(1) = 6.462, *p* = 0.011). Consequently, when a mark was allocated for each correct answer provided (allowing for a maximum correct responses score of 12), radiology practitioners achieved a mean score of 7.42 (SD 2.28) while radiographers achieved a mean score of 7.02 (SD 1.56), but this difference was not found to be statistically significant (Mann–Whitney, *p* = 0.288). Nonetheless, *χ*^2^ test analysis did reveal a strong association between the total ‘correct responses score’ and the participants’ profession, *χ*^2^(9) = 20.265, *p* = 0.016, which is probably linked to the higher percentage of radiology practitioners (75 %) at least scoring 8/12 when compared to the 35 % of radiographers who managed to do so (Fig. 3). No participant obtained the maximum score of 12, although one radiology practitioner and one radiographer did score 11/12.

### Use of referral guidelines

The majority of radiology practitioners and radiographers claimed that they did not make use (31.0 %, 34/110) or were ‘not sure’ (46.3 %, 51/110) of the use of referral guidelines/criteria for paediatric MI examinations. Furthermore, from the 22.7 % (25/110) who indicated that they did make use of referral criteria/guidelines, only 12 participants (12/110, 10.9 %) were able to name the Radiation Protection 118 referral guidelines document, which had been established as the official referral guidelines for imaging examinations at the hospital studied, in accordance with national and European regulations [7, 8]. Consequently, three participants (2.7 %) mentioned the European Association of Nuclear Medicine (EANM) guidelines for paediatric examinations while another two (1.8 %) mentioned the Royal College of Radiologists (RCR) iRefer Guidelines.

### Consideration of previous radiological imaging examinations

Approximately four in five participants (78.2 %, 86/110) indicated that they ‘very often’ or ‘always’ consider previously undertaken radiological examinations prior to performing another MI examination on the same paediatric patient (Table 3). This response potentially highlights the radiology practitioners’ and radiographers’ active role in abiding to their ‘clinical responsibility’ for the medical exposure as outlined by European and national legislation [7, 8], whereby imaging practitioners are expected to seek information about previous examinations performed.

**Table 3:** Responses to the question: ‘For a paediatric patient, how often would you generally consider previous radiological examinations undertaken, prior to performing another medical imaging examination for that same child?’

	Profession		
	Radiology practitioners	Radiographers	Total
Never	0	3	3 (2.7 %)
Rarely	1	6	7 (6.4 %)
Sometimes	1	13	14 (12.7 %)
Very Often	1	34	35 (31.8 %)
Always	9	42	51 (46.4 %)
**Total**	**12**	**98**	**110 (100 %)**

*X* ^2^(4) = 5.371, *p* = 0.251

## Discussion

To our knowledge, this is the first study providing a comprehensive insight into radiology practitioners’ and radiographers’ level of awareness concerning radiation doses typically associated with paediatric MI examinations. Consequently, it is the first study exploring the use of referral guidelines and the level of awareness of paediatric imaging radiation doses amongst imaging practitioners in Malta.

While comparison of results is rather limited, since previously published studies did not solely focus on paediatric imaging examinations and did not make use of an identical research design or questionnaire, it is evident that the findings of this study are consistent in reporting a poor level of radiation dose awareness amongst health professionals [13–24]. In fact, on average, only 20 % of the participating radiology practitioners and radiographers were aware of the estimated ED for five paediatric radiation-based MI examinations, with the percentage of correct ED estimations varying from 9.6 to 32.4 %. Furthermore, while the majority were aware that MRI and ultrasound did not use ionising radiation, two radiology trainees and sixteen radiographers did not know this or else allocated an ED for such examinations. While it is possible that the radiology trainees incorrectly answered this question as they were still in the early stage of their training, we cannot explain why so many radiographers got this wrong. Consequently, although these findings are consistent with what has been reported previously for medical physicians, paediatricians, and surgeons [16, 18, 21–24], such a lack of awareness amongst imaging practitioners raises some concern, particularly in view of the important role they may have in the justification of paediatric MI examinations, whereby the use of ultrasound or MRI should be considered and encouraged when such examinations are likely to provide the necessary diagnostic information within a reasonable time.

When considering previously published research, two studies were found asking their respondents to provide an ED estimate for a paediatric chest CT [16, 24] and a paediatric abdominal CT. Just over a third (35 %) of participating paediatricians and 21.7 % of participating paediatricians, surgeons, and general practitioners respectively provided a correct ED estimate for the paediatric CT examination [16, 24]. Consequently 40.3 % of participating paediatricians, surgeons, and general practitioners correctly provided the dose estimate for the paediatric abdominal CT examination [24]. In comparison, 24.5 and 21.2 % respectively provided the correct ED estimate for the paediatric chest and abdominal CT examinations in this study, despite the fact that one would probably expect radiology practitioners and radiographers to demonstrate a better understanding than other health professionals in this regard. Consequently, most of our study’s findings seem to be consistent with those reported in other studies, which assessed radiation dose awareness of different adult MI examinations. Indeed, the 21.2 % who correctly estimated the ED for an abdominal CT scan (5 year old) in this study are similar to those reported by Lee et al., whereby 22 % of physicians and 15 % of radiologists correctly estimated the ED of an adult abdominal CT scan [19]. Moreover, these findings are better than those reported in two previously published UK studies, within which only 7–8 % of participating physicians respectively provided the correct ED estimate for an abdomen CT scan and for the foetal dose for a CT pulmonary angiography examination [20, 34].

More than a third (34.3 %) of participants overestimated the associated ED for a paediatric CT head scan, which is the most common CT examination performed in paediatric patients; while another 27.5 % and 24.0 %, respectively, overestimated the ED typically associated with CT thorax and CT abdomen scans performed on a 5-year-old. In the absence of local diagnostic reference levels for paediatric CT examinations, we believe that these findings are significant because, if radiology practitioners and radiographers incorrectly perceive the ED of these common paediatric CT scan examinations to be higher than they typically should be, this may limit them from identifying those examinations which are yielding an excessive dose and that require child-sizing (optimisation) of scan parameters and/or technique.

Conversely, about 24 % of responses in this study related to an underestimated ED. While this was much lower than that reported by Shiralkar et al. whose study sample of 130 doctors (including ten consultant radiologists) underestimated 97 % of the actual dose of various adult MI examinations [21], we feel that it is still an important finding. Indeed, underestimation of dose may not only lead to an incorrect perception of the risks involved, but it may also precondition the imaging practitioner in believing that, since the ED is low, the need to optimise such an examination is also low. Furthermore, since nearly half (48.0 %) of the participating imaging practitioners underestimated the ED associated with an abdominal CT scan performed on a 1-year-old patient, we believe that this may reflect imaging practitioners’ lack of consideration of the fact that younger paediatric patients receive a much higher ED per unit of radiation when compared to adults undergoing the same examination. In addition, when considering that 78.3 % either did not know or else underestimated the potentially high ED of a fluoroscopically-guided coronary angiography intervention, one may question whether imaging practitioners involved in such procedures are attentive to optimise their technique and exposure parameters so as to reduce the possibility for adverse tissue reactions and stochastic effects.

It was surprising to note that only one statistically significant difference was found in the responses provided by radiology practitioners and radiographers, although we do recognise that the small sample of radiology practitioners may have contributed to this result. This difference related to the statement concerning the principle of optimisation, whereby 95 % of radiographers correctly recognised that consideration of radiation dose is an important aspect of optimisation in comparison to the 75 % of radiology practitioners. While this may reflect radiographers’ active role in the optimisation of MI examinations, it is slightly concerning to note that 25 % (*n* = 3) of radiology practitioners were of the opinion that each medical exposure should produce the best imaging quality for diagnosis. Indeed, this finding raises questions as to the potential effect this particular mindset can have in practice, particularly in view of radiology practitioners’ input into imaging protocols, as well as them requesting that an imaging examination needs to be repeated.

Consistent with findings of a report published by the European Commission [26], the majority of participating radiology practitioners and radiographers were not aware or else did not use referral guidelines. This was a rather unexpected finding since each staff member had received an internal circular when the RP118 document was established as the official referral guidelines for all imaging examinations at the hospital during the previous year [35]. Nonetheless, as recognised in the EU report, additional measures are needed on both a European and national levels to reinforce the use of guidelines, particularly since they are specifically designed to help health professionals in deciding the most appropriate imaging examinations for given clinical indications/scenarios. Furthermore the literature suggests that the use of referral guidelines has the potential to bring about a 13–20 % reduction in referral rates, which in turn may lead to a potential dose saving for patients [25]. Therefore, coupled with the poor level of awareness concerning radiation doses demonstrated by this study’s participants, it is recommended that all local radiology practitioners and radiographers are not only made aware of that such guidelines exist, but they should also be educated and trained on how to make effective use of them during the justification process as well as in their discussions with referring physicians and patients.

Half of the radiology practitioners and radiographers reported that they had undertaken a maximum of 20 h of radiation protection education and training, which is much less than the recommended 30–50 h for radiology practitioners and 100–140 h for radiographers [36]. While this may possibly be true for radiology practitioners who only receive radiation protection education and training during their postgraduate studies and/or radiology specialisation, it does not reflect the number of hours most radiographers perform as part of their undergraduate radiography course programme. For this reason, we believe that participants may have underreported the amount of hours of radiation protection education and training received, possibly by overlooking a number of topics that are interrelated to physiological/pathological processes and or radiology/radiography principles. Nonetheless, given that a considerable number of participants indicated that they had not undertaken radiation protection education/training for at least 5 years, it is important that imaging practitioners to recognise the importance of remaining up to date with the latest techniques, devices and software that can contribute to considerable radiation dose savings for their patients and fellow colleagues.

### Strengths and limitations

The questionnaire used for the study was designed following a thorough process that assessed and verified its reliability and validity. We believe that it is also the first questionnaire to specifically explore the level of radiation dose awareness of paediatric imaging examinations amongst radiology practitioners and radiographers. The 66.7 % response rate obtained in this study was quite satisfactory, although we must also acknowledge that the responses provided may not necessarily be representative of the entire population of radiology practitioners and radiographers working at this primary paediatric referral centre in Malta. Nonetheless, we do believe that our study sample is comparable to the target population of imaging practitioners, particularly since the characteristics represented in our study sample are consistent with those of the relatively young workforce of radiology practitioners and radiographers at the hospital studied. Furthermore we also recognise that the use of questionnaires has its own limitations, with the possibility that some participants may not have been truthful in responses concerning their opinion, perception, or actual practice. We also recognise that participants had the opportunity to refer to textbooks and/or internet resources to complete the questionnaire. Nonetheless, given the busy work schedules that both radiology practitioners and radiographers generally have, we believe that it is unlikely that many of the participants would have taken the time to search for the most appropriate responses for the questions posed in our questionnaire.

## Conclusion

In conclusion, local imaging practitioners’ limited use of referral guidelines and lack of radiation dose awareness highlights a potential gap in knowledge that may impede their role in the justification and optimisation of paediatric imaging examinations. Furthermore, if radiology practitioners and radiographers do not fully understand the relative radiation levels associated with paediatric imaging examinations, this not only limits their ability to communicate accurate benefit-risk information to the paediatric patients and their parents, but it also restricts informed discussions between the imaging practitioners and referring physicians. For these reasons, the implementation of regular radiation protection education and training post qualification activities are encouraged so as to ensure that local imaging practitioners are able to fulfil their roles as effectively as possible while ensuring the best and safest practice for all patients.

## Abbreviations

ED: Effective dose
MI: Medical imaging
